# Viral Hepatitis B and C: Knowledge Gaps and Patterns of Preventive Practices Among Medical Doctors in Rivers State, Nigeria

**DOI:** 10.7759/cureus.22928

**Published:** 2022-03-07

**Authors:** Sarah Abere, Boma Oyan, Dan-Jumbo Alali, Hannah Omunakwe, Mazpa Ejikem

**Affiliations:** 1 Internal Medicine, Rivers State University Teaching Hospital, Port-Harcourt, NGA; 2 Haematology, Rivers State University Teaching Hospital, Port-Harcourt, NGA; 3 Internal Medicine, University of Port-Harcourt Teaching Hospital, Port-Harcourt, NGA

**Keywords:** universal precaution, personal protective equipment, hcv, hbv, viral hepatitis b and c, healthcare workers

## Abstract

Introduction

Hepatitis B and C viruses can result in both acute and chronic hepatitis, ranging in severity from a mild acute disease to a serious, lifelong manifestation including liver cirrhosis and hepatocellular carcinoma. This study aims to determine the knowledge and attitudes of medical doctors in Rivers State, Nigeria, to hepatitis B and C as well as their practice for prevention and treatment of the same. We also assessed their practice of universal precaution, provision, and use of personal protective tools.

Methods

One hundred and fifty doctors in both the private and public sectors were interviewed using self-administered questionnaires on viral hepatitis B and C. The questionnaire explored their knowledge and status of vaccination, viral hepatitis treatment, and their practice of universal safety precautions. Their responses were analyzed using SPSS version 21. Data was expressed in means and percentages.

Results

Out of all participants, 96% were aware that viral hepatitis B is preventable, while 46% erroneously believed that there is vaccination against the hepatitis C virus. Only 50% of the respondents were aware of the availability of a cure for hepatitis C infection, and 16% of the participants knew about drugs used for its treatment. While 76% of the doctors had been vaccinated against hepatitis B virus, only 4% had received treatment after testing positive for hepatitis B. Furthermore, nearly all respondents admit practicing universal precaution, especially during venipuncture; however, protective measures such as disposable gloves were not readily available to 20% of our respondents. There was a statistically significant association between sex and duration of practice with knowledge of hepatitis B and C, as well as between practice type and vaccination status.

Conclusion

This study shows that knowledge of the treatment of viral hepatitis amongst healthcare practitioners such as doctors is poor, and although universal safety precautions are practiced, personal protective equipment is not readily available for use in our healthcare setting, placing healthcare workers at risk of infections. There is also a need to encourage vaccination amongst healthcare practitioners to protect them against contagious diseases like hepatitis B and C infections.

## Introduction

Hepatitis describes the inflammation of the liver. It could be caused by a variety of factors, including infections, alcohol, drugs, metabolic diseases, autoimmune disorders, etc. Viral hepatitis refers to the inflammation of the liver due to viral infections by one or more of the hepatitis viruses, including hepatitis A, B, C, D, or E viruses, and infrequently by cytomegalovirus, adenoviruses, Epstein-Barr virus, and herpes simplex virus. Viral hepatitis B and C (HBV and HCV, respectively) can cause acute infections, which may result in outcomes ranging from self-limiting hepatitis to liver fibrosis, cirrhosis, and hepatocellular carcinoma.

According to the World Health Organization (WHO), approximately 257 million people live with chronic HBV infection, 71 million people with chronic HCV infection globally, and an estimated 1.34 million people around the world died from viral hepatitis in 2015, a number comparable to deaths caused by tuberculosis and higher than those caused by HIV [1]. 

In Africa, 60 million people and 11 million people live with hepatitis B and C [1]. The prevalence of viral hepatitis B and C in Nigeria is 11% and 2.2%, respectively, with an average prevalence of HBV that ranges between 11- 13.7%, with an estimation of 20 million Nigerians chronically infected [2]. The global epidemic of viral hepatitis is 10 times higher than that of HIV, and chronic viral hepatitis is now the second-largest killer infection after tuberculosis [3].

The hepatitis B virus (HBV) is a double-stranded DNA virus of the Hepadnaviridae family of viruses. The hepatitis C virus (HCV) is a single-stranded RNA virus of the Flaviviridae family. Humans are the only known natural host to hepatitis B and C viruses, and the viruses circulate in the blood in concentrations as high as 108 virions per ml. Transmission of HBV and HCV occurs through percutaneous or per mucosal exposure to infective body fluids, sexual contact, and drug injection; nosocomial transmission has also been documented [4].

Many workers are frequently exposed to risk factors associated with their professional activities, and healthcare workers (HCWs) are not an exception. HCWs, especially doctors and nurses, represent a high-risk population for HBV and HCV infection. HBV is said to be the most infectious occupational hazard seen among HCWs [5]. Burzoni et al. reported that biological risk in the workplace is responsible for over 300,000 deaths per year worldwide [6]. This underlines the importance of increased awareness of the existence of these agents and the need for personal protection practices. Exposure to biological fluids such as urine, blood, and blood products, semen/vaginal fluids, and cerebrospinal fluids are sources of contamination, especially for viral infections [7]. Many HCWs in the course of their duties are exposed to hepatitis B and C infection through percutaneous transmission following a needle stick injury or cuts with infected sharp objects.

The knowledge and awareness of viral hepatitis in healthcare practitioners varies according to their level of education [8]. Mehriban et al. reported that 67.3% of the HCWs had an adequate level of knowledge on hepatitis B, but only half of them (49.3%) had a good level of preventive practices [5]. It has been observed that healthcare workers have inadequate knowledge of how to protect themselves from blood-borne diseases, and this inadequacy could lead to low adherence to safety measures among them.

Lack of awareness of HBV and HCV modes of transmission, prevention, and treatment would probably contribute to the continuing transmission, exposure, and subsequent infection of HCWs, as well as missed opportunities for prevention (including vaccination) and treatment of infected persons.

In a knowledge and awareness survey of the American National Strategy for Prevention and Control of HBV and HCV, it was concluded that the knowledge about chronic HBV and HCV among HCWs was generally poor and insufficient [9]. Furthermore, a study conducted in 2009 among 1,362 Chinese non-specialist physicians on the knowledge of HCV infection reported that the respondents had little understanding of HCV infection, which might hamper their care of HCV patients [10]. In addition, despite the increased risk of exposure to biological agents faced by HCWs, knowledge and self-reported compliance to recommended personal protective equipment (PPE) use among clinicians remains suboptimal [11].

Therefore, we sought to assess the knowledge of hepatitis B and C virus, including their transmission, prevention, post-exposure prophylaxis, and treatment among medical doctors, a subset of healthcare workers in Rivers State, and furthermore to evaluate the percentage of doctors that have been vaccinated against viral hepatitis B and those who have been previously exposed and/or treated for viral hepatitis. Also, we sought to assess the attitudes and compliance to basic universal standard precaution guidelines for personal protection of this subset of healthcare providers in the state.

## Materials and methods

Study design

Rivers State is one of the thirty-six states of Nigeria, located in the southernmost part of the country. There are five tertiary and fifty-four secondary coded healthcare facilities in the state [12]. This study was a descriptive, cross-sectional study conducted among medical doctors in Rivers State, Nigeria.

Sampling methodology

Of the five tertiary health facilities, two of them were specialized hospitals for neuropsychiatry and dental/maxillofacial conditions, and the third (Niger Foundation Hospital) was temporarily closed and were all excluded from the study. The remaining two tertiary hospitals were then chosen, including the University of Port-Harcourt Teaching Hospital and Rivers State University Teaching Hospital. Out of the fifty-four secondary health facilities, ten hospitals were chosen by convenience sampling methodology, and questionnaires (see appendix) were distributed among 150 medical doctors in these facilities over four months.

Data collection

Data was collected using a structured, self-administered questionnaire. Fully informed and appropriate consent of the participants was sought and obtained. Demographics, including age, gender, years of practice, and private or public practice types, were assessed. Also, the questionnaire explored the knowledge of the participants on transmission, prevention, and drug treatment of hepatitis B and C, as well as their personal vaccination and treatment history on hepatitis B and C. Each correct response to the knowledge questions was scored one mark, and any wrong or non-response was scored zero. The total score obtained by each respondent was converted to a percentage and graded as poor (<50%), fair (50-74%), and good (≥75%) [13].

Data analysis

Data was assessed using a Statistical Package for Social Sciences (SPSS) version 21 (IBM Inc., Armonk, USA). Data was expressed as frequencies in percentages. Continuous variables were compared with the Student's t-test, while categorical parameters were compared with a chi-squared test. A p-value of ≤0.05 was considered statistically significant.

Ethics

Ethical approval for the study was obtained from Rivers State University Teaching Hospital Ethics Committee (number: RSUTH/REC/2022153). Informed consent was sought and obtained from respondents before administering a questionnaire.

## Results

Socio-demographic characteristics

A total of one hundred and fifty (150) doctors were assessed via structured questionnaires over a four-month period. The male to female ratio was 1.08. The modal group for years of practice was 0-10 years and 132 (88%) of the respondents work in a public/government facility while 18 (12%) were working in private facilities. (Table 1).

**Table 1:** Socio-demographic characteristics of the study population (n=150)

Characteristic	Frequency (%)
Sex	
Male	78 (52.0)
Female	72 (48.0)
Duration of practice (years)	
0 - 10	54 (36.0)
10 - 20	15 (10.0)
20 - 30	15 (10.0)
30 - 40	30 (20.0)
>40	36 (24.0)
Practice type	
Public	132 (88.0)
Private	18 (12.0)

Knowledge of prevention, curability and mode of transmission

**Figure 1:**
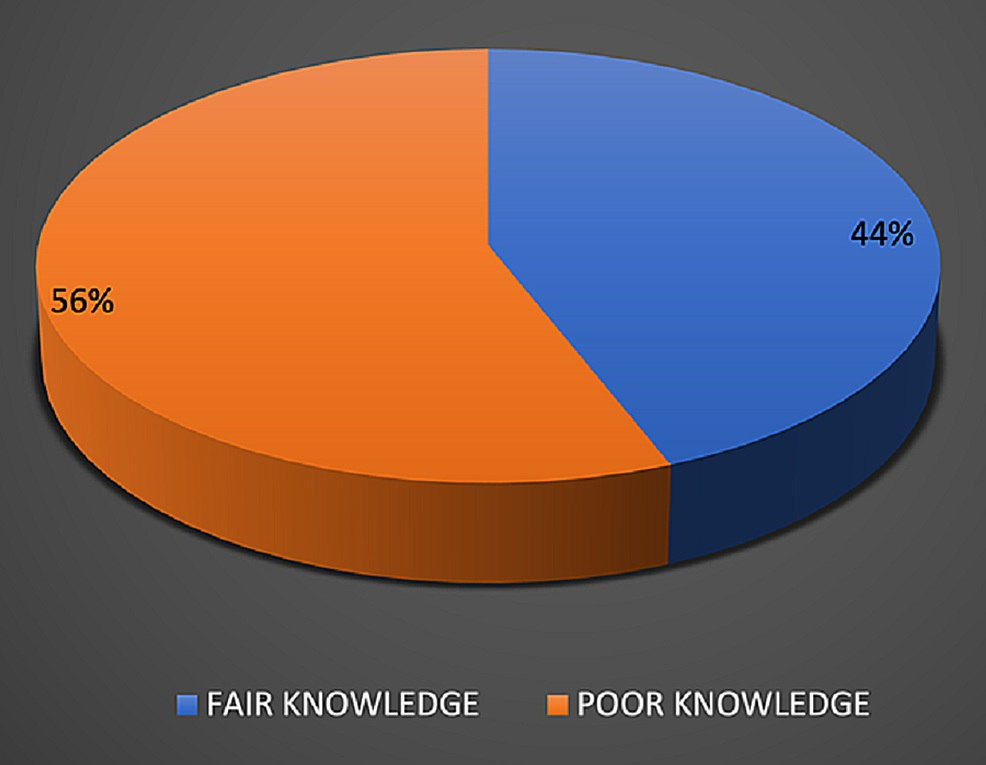
Knowledge scores on hepatitis B and C in the study population

None of the respondents were found to have good knowledge, with 44% of doctors having fair knowledge and more than half of the doctors (56%) showing poor knowledge on hepatitis B and C (Figure 1).

As shown in Table 2, a large majority (96%) of the doctors assessed believed that HBV infection is preventable by vaccination while 46.0% believed HCV infection is preventable by vaccination. While 42% believed HBV infection can be cured, half of the respondents believed there was no cure for HCV infection. Concerning modes of transmission of both HBV and HCV, 84% of the respondents knew that vertical transmission occurred with HBV while 36% knew same for HCV. Also, 96% of respondents believed that HBV is transmissible by needle prick injury/blood products while 46.6% of respondents were aware that HCV is transmissible by needle prick injury/blood products. Most of the respondents (78%) were aware of post exposure prophylaxis for viral hepatitis, and 86% of the respondents were aware of booster vaccination for HBV. However, majority of respondents were not aware of the drug treatments for HCV, including ledipasvir (94%), simeprevir (94%), daclatasvir (92%), with ribavirin (26%) and interferon (36%) being the drugs for treatment of HCV most known to the respondents. Interferon (68%) and lamivudine (54%) were the drugs most known to be useful for treating HBV; however, 78% and 92% of respondents were not aware of the use of tenofovir and entecavir, respectively, for the treatment of HBV infection (Table 2).

**Table 2:** Knowledge on viral hepatitis B and C in the study population (n=150) HBV - hepatitis B virus; HCV - hepatitis C virus

Variable	Yes (%)	No (%)
HBV is preventable by vaccination	144 (96.0)	6 (4.0)
HCV is preventable by vaccination	69 (46.0)	81 (54.0)
HBV is curable	63 (42.0)	87 (58.0)
HCV is curable	75 (50.0)	75 (50.0)
Commoner virus		
HBV	144 (96.0)	-
HCV	6 (4.0)	-
Knowledge of modes of transmission of HBV		
Needle-prick injury	144 (96.0)	6 (4.0)
Vertical transmission	126 (84.0)	24 (16.0)
Pleural fluids	108 (72.0)	42 (28.0)
Urine	81 (54.0)	69 (46.0)
Amniotic fluid	90 (60.0)	60 (40.0)
Knowledge of modes of transmission of HCV		
Needle-prick injury	69 (46.0)	81 (54.0)
Vertical transmission	54 (36.0)	96 (54.0)
Pleural fluids	36 (24.0)	114 (76.0)
Urine	30 (20.0)	120 (80.0)
Amniotic fluid	45 (30.0)	105 (70.0)
Knowledge of drug treatment for viral hepatitis		
Awareness of post-exposure prophylaxis	117 (78.0)	33 (22.0)
Awareness of booster vaccination for HBV	129 (86.0)	21 (14.0)
Lamivudine for HBV	81 (54.0)	69 (46.0)
Interferon for HBV	102 (68.0)	48 (32.0)
Tenofovir for HBV	33 (22.0)	117 (78.0)
Entecavir for HBV	12 (8.0)	138 (92.0)
Ribavirin for HBV	42 (28.0)	108 (72.0)
Ledipasvir for HCV	9 (6.0)	141 (94.0)
Simeprevir for HCV	9 (6.0)	141 (94.0)
Daclatasvir for HCV	12 (8.0)	138 (92.0)
Ribavirin for HCV	39 (26.0)	111 (74.0)
Interferon for HCV	54 (36.0)	96 (64.0)
Sofosbuvir for HCV	24 (16.0)	126 (84.0)

There was a significant association between knowledge scores and the sex and duration of practice of the respondents with males having higher knowledge scores (p=0.011) and doctors with a duration of practice between 0 and 10 years reporting higher knowledge scores (p=0.003; Table 3).

**Table 3:** Association between socio-demographic characteristics with knowlege on hepatitis B and C (n= 150) * Significant p-value

Variable	Good	Fair	Poor
Sex			
Male	-	42	36
Female	-	24	48
	X^2^=6.394	Df=1	p=0.011*
Duration of practice			
0-10	-	18	36
10-20	-	12	3
20-30	-	6	9
30-40	-	9	21
>40	-	21	15
	X^2^=15.869	Df=4	p=0.003*
Practice type			
Public	-	60	72
Private	-	6	12
	X^2^=0.945	df=1	p=0.331
Vaccination status			
Yes	-	51	63
No	-	15	21
	X^2^=0.105	df=1	p=0.746

Pattern of prevention and management practices of viral hepatitis B and C 

As shown in Table 4, 76% of the doctors had been vaccinated against the hepatitis B virus. While all the respondents used hand gloves during venepuncture and sample collection, only 80% of the respondents used gloves during physical examination, and 94% had gloves available at their practice centre. Only 64% of respondents always practiced handwashing before and after procedures. While no respondent had been treated for HCV infection, six (4%) of them had been diagnosed with HBV infection and had all received treatment for HBV infection. Nearly three-quarters (74%) and 34% of the respondents had been involved in the treatment of a patients with HBV and HCV infection, respectively.

**Table 4:** Pattern of prevention and management practices of viral hepatitis B and C in the study population (n=150) HBV - hepatitis B virus; HCV - hepatitis C virus

Variable	Yes (%)	No (%)
Have you been vaccinated for HBV?	144 (76.0)	36 (24.0)
Are gloves available at your centre?	141 (94.0)	9 (6.0)
Use of gloves during physical examination	120 (80.0)	30 (20.0)
Use of gloves for venepuncture and sample collection	150 (100.0)	0 (0.0)
Practice of handwashing pre and post procedures		
Sometimes	54 (36.0)	-
Always	96 (64.0)	-
Previous treatment		
HBV	6 (4.0)	144 (96.0)
HCV	0 (0.0)	150 (100.0)
Treated patients		
HBV	111 (74.0)	39 (26.0)
HCV	51 (34.0)	99 (66.0)

A significant association was noted between the type of practice and the HBV vaccination status, with those in public practice being more vaccinated than those in private practice (Table 5). This association may be attributable to better access to vaccines in public hospitals.

**Table 5:** Association between socio-demographic characteristics and vaccination status against viral hepatitis B (n=150) * Significant p-value

Variable	Yes	No	p-value
Sex			
Male	60	18	
Female	54	18	
	X^2^=0.076	df=1	p=0.783
Duration of practice			
0-10	39	15	
10-20	12	3	
20-30	12	3	
30-40	27	3	
>40	24	12	
	X^2^=5.629	df=4	p=0.229
Practice type			
Public	105	27	
Private	9	9	
	X^2^=7.581	df=1	p=0.006*

## Discussion

This study aimed to assess the knowledge of HBV and HCV among doctors working in both private and public healthcare settings in Rivers State, Nigeria, in several domains, including transmission, prevention, prophylaxis, and treatment modalities. Vaccination status, exposure, and treatment for viral hepatitis were also evaluated, as well as compliance and challenges to the practice of universal, standard precautions for personal protection.

The high level of awareness for vaccination against HBV (96%) was seen among the respondents in this study is similar to that reported in several other studies that reported awareness rates as high as 87%, 92%, and 96%, respectively [14-16].

Our study reported a low rate of knowledge (46.6%) of the routes of transmission of HCV. This corresponds with findings in a study on the knowledge and attitude of healthcare workers towards HCV, where deficiencies range from 48% to 68% [17].

In our study, males reported a significantly higher proportion of respondents with better knowledge of hepatitis B and C as compared to females. This contrasts with previous studies which report better knowledge levels among females on hepatitis B [18] and C [19,20].

It is imperative to note that misinformation about HBV transmission and treatment among doctors, as observed in this study, may create obstacles against prevention and treatment. Only 42% of our respondents were aware of close household contacts as an established high-risk source of transmission of HBV, necessitating screening as the risk of chronic infection after exposure to HBV is highest in early life. A cross-sectional survey conducted among 217 members of the New Jersey Academy of Family Physicians similarly reported that only 50% of survey participants recommended screening household contacts of persons who had chronic HBV infection - an established high-risk population [21].

The majority of our respondents (76%) have been vaccinated against HBV, and 86% of them were aware of the need for a booster dose. In addition, the knowledge on post-exposure prophylaxis was significantly different among participants based on the duration of practice.

Fewer doctors (34%) have been involved in the management of HCV patients despite the rising prevalence of HCV in our environment, and this may be explained by a dearth in the knowledge of the recent drugs available for its treatment; ≤16% of the participants were aware of the direct-acting antivirals for HCV treatment. This highlights the need for continuous medical training of doctors, especially with the constantly changing landscape of HCV treatment.

Few doctors reported awareness of tenofovir, a cheap, effective, and readily available nucleoside analog, as a drug treatment for HBV and this low awareness was significantly higher among older practitioners. Only 12 of the respondents were aware of the drug entecavir - another effective nucleoside for HBV therapy. Considering the high prevalence of HBV in Nigeria, low knowledge of HBV treatment among doctors could negatively impact the drive towards the elimination of viral hepatitis by 2030 [22] and the sustainable development goal of increasing treatment for viral hepatitis from 1% to 80% by the year 2030 [23].

It is important to note that only about half (54%) and two-thirds (68.0%) of the participants were aware of lamivudine and interferon as drugs used for HBV treatment. The knowledge on lamivudine, tenofovir, entecavir, and ribavirin was significantly correlated with the duration of practice among the respondents, while no significant association was found between duration of practice and knowledge on interferon as drug treatment for HBV.

From our study, interferon was the only HCV drug known to a substantial proportion of our respondents (33.3%), closely followed by ribavirin (22.2%), which did not seem to depend on years of practice. However, there is low knowledge of newer and better drugs available for HCV, which reflects a need for continued training of non-gastroenterology doctors to manage viral hepatitis patients. Dublin et al. reported a similar knowledge gap in HCV treatment and curability among primary care providers compared to GI specialists [24]. A survey of 1,412 primary care providers in the United States that assessed knowledge about risk factors for HCV infection and management of hepatitis C reported that 27% of their respondents did not know which therapy to use after a patient is diagnosed HCV positive [24]. Furthermore, doctors who had practiced for more than 30 years had lower knowledge of newer HCV treatment drugs, which is similar to findings reported by Shehab et al. [25,26].

Nearly half of the respondents in this study erroneously believed there is a curative treatment for HBV, and this corresponds with findings of a study conducted by Wu et al., which reported that 46% of their study population incorrectly responded that chronic HBV infection is curable [27]. This further supports the need for continued medical education of healthcare providers involved in viral hepatitis care.

Our study was limited by the relatively low number of respondents who participated in the study. Also, our study should have been carried out over a longer duration and should have a pre- and post-training component which could have assessed the knowledge of the participants better.

## Conclusions

There is a high prevalence of viral hepatitis B and C in Africa. Our study, however, showed that knowledge about viral hepatitis B and C amongst medical doctors practicing in Rivers State, a highly populated Nigerian state, remains poor, especially in the domains of prevention and drug treatment for hepatitis B and C. There is, however, relatively good knowledge on universal safety precautions. Knowledge scores were found to be significantly correlated with the respondents' sex, duration of practice, as well as type of practice. While a large majority of medical doctors had access to personal protective equipment, there was a limited practice of universal safety precautions among them. There is also a need to encourage vaccination amongst doctors to prevent HBV infection.

## Appendices

Questionnaire

Introduction

My name is Dr. Sarah Abere and I lead a team of medical researchers to conduct a study titled: “Viral Hepatitides B and C: Knowledge Gaps and Patterns of Preventive Practices Among Medical Doctors in Rivers State”. We will be interviewing medical doctors in Rivers State using this questionnaire. The objective of this study is to determine the knowledge and preventive practices of medical doctors on hepatitis B and C.

Confidentiality and Consent

During this study, you will be asked questions and your responses are going to be completely confidential and will not be used for any other purpose other than the above study. Your name is not going to be written on this form and thus, will not be used in connection with the information you provide. Please do NOT write your name. You are hereby informed that you do not have to answer any questions that you do not want to answer. However, we will appreciate your honest and appropriate answers in response to this study. There is no risk associated with your involvement in this study.

Your participation in this research will only require about 5 minutes of your time. Only numbers will be given to all completed questionnaires and names will not be recorded. Finally, your participation in this research is entirely voluntary.

Statement of Consent

I have read the description of this research and I understand it. I also understand that my participation is voluntary. I know enough about its objectives, methods, risk and benefits of the research study and I ascertain that I want to participate in it. I am duly informed that I may choose to stop responding to the questionnaire at any time I feel so.

Signature of participant/date : _______________________________________________________________________________

**Table 6:** Questionnaire on viral hepatitides B and C - knowledge gaps and patterns of preventive practices among medical doctors in Rivers State HBV - hepatitis B virus; HCV - hepatitis C virus

Socio-demographic characteristics					
How old are you (in years)?				__________________	
What is your sex?				( ) Male	( ) Female
How long have you been practicing medicine (in years)?					
( ) 0 – 10	( ) 11 – 20	( ) 21 – 30	( ) 31 – 40	( ) > 40	
Type of practice				( ) Public	( ) Private
Knowledge on hepatitis B and C				HBV	HCV
Which is the commoner virus (please choose one option only)				( )	( )
Which of the viruses is preventable				( )	( )
Which of the viruses are curable				( )	( )
Vertical transmission is commoner in				( )	( )
Exposure to the following can transmit the virus(es)					
Needle prick injury/blood				( )	( )
Vaginal fluids				( )	( )
Pleural fluids				( )	( )
Urine				( )	( )
Amniotic fluid				( )	( )
Are you aware of the following? (Please choose only one option)				Yes	No
Post-exposure prophylaxis for HBV				( )	( )
Booster vaccination for HBV?				( )	( )
Which of the following drugs is/are useful in treatment of HBV? (Please select all that apply)					
( ) Lamivudine	( ) Interferon	( ) Tenofovir	( ) Entacavir	( ) Ribavirin	
Which of the following drugs is/are useful in treatment of HCV? (Please select all that apply)					
( ) Ledipasvir	( ) Simeprevir	( ) Daclatasvir	( ) Ribavirin	( ) Interferon	( ) Sofosbuvir
Pattern of prevention and management practices				Yes	No
Have you been vaccinated for HBV?				( )	( )
Are hand gloves available at your centre?				( )	( )
Do you use gloves during physical examinations?				( )	( )
Do you use gloves for venipuncture and sample collection?				( )	( )
Have you ever been treated for HBV?				( )	( )
Have you ever been treated for HCV?				( )	( )
Have you ever treated a patient with HBV?				( )	( )
Have you ever treated a patient with HCV?				( )	( )
How would you describe your practice of handwashing before and after procedures?					
( ) I don’t bother		( ) Sometimes		( ) Always	
